# Nutrient-cycling mechanisms other than the direct absorption from soil may control forest structure and dynamics in poor Amazonian soils

**DOI:** 10.1038/srep45017

**Published:** 2017-03-23

**Authors:** Oriol Grau, Josep Peñuelas, Bruno Ferry, Vincent Freycon, Lilian Blanc, Mathilde Desprez, Christopher Baraloto, Jérôme Chave, Laurent Descroix, Aurélie Dourdain, Stéphane Guitet, Ivan A. Janssens, Jordi Sardans, Bruno Hérault

**Affiliations:** 1 CSIC, Global Ecology Unit CREAF-CSIC-UAB, 08193, Cerdanyola del Vallès, Catalonia, Spain; 2 CREAF, 08193, Cerdanyola del Vallès, Catalonia, Spain; 3 AgroParisTech, ENGREF, UMR, 54000, Nancy, France; 4 CIRAD, UR Forêts et sociétés, 34398, Montpellier, France; 5 CIRAD, UR Forêts et sociétés, 34398, Montpellier, France; 6 CIRAD, UMR Ecologie des Forêts de Guyane, 97387, Kourou, French Guiana, France; 7 International Center for Tropical Botany, Department of Biological Sciences, Florida International University, 11200, Miami, USA; 8 CNRS, Laboratoire Evolution et Diversité Biologique, 31062, Toulouse, France; 9 ONF Guyane, Réserve de Montabo, 97307, Cayenne, French Guiana, France; 10 INRA-ONF, UMR Amap, 34398, Montpellier, France; 11 University of Antwerp, Department of Biology, 2610, Wilrijk, Belgium

## Abstract

Tropical forests store large amounts of biomass despite they generally grow in nutrient-poor soils, suggesting that the role of soil characteristics in the structure and dynamics of tropical forests is complex. We used data for >34 000 trees from several permanent plots in French Guiana to investigate if soil characteristics could predict the structure (tree diameter, density and aboveground biomass), and dynamics (growth, mortality, aboveground wood productivity) of nutrient-poor tropical forests. Most variables did not covary with site-level changes in soil nutrient content, indicating that nutrient-cycling mechanisms other than the direct absorption from soil (e.g. the nutrient uptake from litter, the resorption, or the storage of nutrients in the biomass), may strongly control forest structure and dynamics. Ecosystem-level adaptations to low soil nutrient availability and long-term low levels of disturbance may help to account for the lower productivity and higher accumulation of biomass in nutrient-poor forests compared to nutrient-richer forests.

Tropical forests play a major role in the global carbon balance, assimilating over a third of the global terrestrial gross primary production^1^. Abiotic factors such as the length of the dry season, precipitation, temperature, and light largely control tree growth in the tropics^2^,^3^. Nevertheless, most tropical forest trees grow in regions with high monthly rainfall (>100 mm; ref. 4) for at least part of the year and usually on old, acidic soils^5^. The leaching of nutrients over a long period consequently drastically lowers the content of nutrients in tropical soils^6^.

The limitation of soil nutrients can strongly control the productivity of tropical forests^7^. Data on phosphorus (P) availability has been crucial for understanding the variations in Amazonian and Bornean forest productivity^8^,^9^. Quesada *et al*.^10^ found a positive correlation between wood production and soil P and nitrogen (N) contents across Amazonian forests, and Aragão *et al*.^11^ found that net primary productivity increased with the statuses of soil P and foliar N contents. These findings support Wieder *et al*.^12^, who predicted that N and P would limit future productivity and carbon-storage capacity. Studies have accordingly highlighted the need to further explore the role of nutrients to improve the accuracy of global carbon-climate models^13^.

Amazonian forest turnover (both recruitment and mortality) is higher on rich soils than on poor soils^10^,^14^, and the growth of tropical trees may be limited by the availability of base cations and/or P (ref. 15). The link between forest aboveground biomass (biomass hereafter) and soil nutrient richness, however, has not been firmly established. Some studies showed that the biomass in tropical forests was higher on nutrient-rich than on nutrient-poor soils^16^,^17^. Other studies, however, found a negative correlation between biomass and amounts of soil cations and P (ref. 10), which may be due to a faster turnover^18^. The number of stems can be lower on the more nutrient-rich soils where low stand densities allow the development of high-biomass trees^19^, suggesting that the relationship between biomass and soil nutrient content is not simple. Furthermore, the importance of litter as a source of nutrients is still disregarded in many studies, despite evidence that litter is an important source of nutrients^20^ and that nutrients may be absorbed directly from the litter by fine roots or mycorrhizae^21^.

Only very few studies have combined data on both forest structure (e.g. tree size, biomass, and stem density) and dynamics (e.g. growth and mortality rates and aboveground wood productivity –productivity hereafter-) from large tropical regions to explore the relationships with soil nutrient contents (e.g. refs 10,11). Our study aims to determine the role of nutrient limitation in the interplay between the structure and dynamics of forests growing on poor tropical soils. We analysed data from French Guiana, a tropical region that has an ancient and nutrient-poor Precambrian geological substrate (Fig. 1, Table S1) that is particularly low in P content (compared to the generally younger, nutrient-rich soils of western Amazonia^7^,^22^. We used the Guyafor network dataset, which contains very precise long-term data on forest structure and dynamics at large (4–12 ha) permanent sites where soil texture and the nutrient contents of soil and litter have been monitored. This dataset offers a unique opportunity to analyse the potential effects of nutrient limitation on the structure and dynamics of the tropical forests of the Guiana Shield. Given that the variables of forest structure and dynamics are expected to be sensitive to small changes in nutrient content in this nutrient-poor ecosystem, we hypothesised that: (1) the variation in nutrient contents of the soil and litter amongst sites would account for most of the variability of forest structure and of forest dynamics across these nutrient-limited ecosystems, (2) higher nutrient content would favour productivity and biomass, and (3) P content would be the most important predictor of the variables of both forest structure and forest dynamics, because P is expected to be particularly limiting. In this study we propose that the structure and dynamics of tropical forests growing on nutrient-limited soils may be governed by alternative mechanisms than those described in tropical regions with richer soils, which could help explain the high accumulation of biomass of forests growing on poor soils.

## Results

### General patterns of forest structure and dynamics and nutrient content

**Figure 1:**
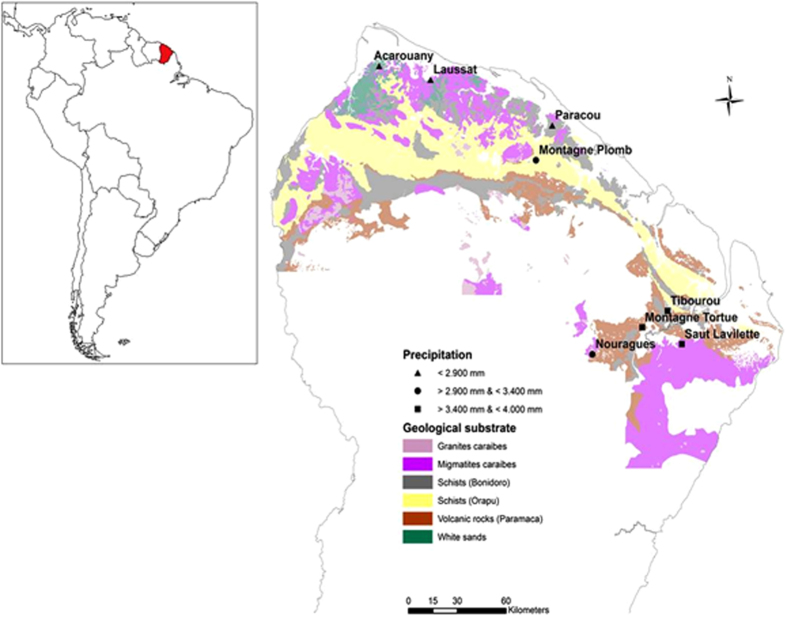
Position of French Guiana in South America (left) and location of study sites (right) within the Guyafor network. The geological substrate and the mean annual precipitation of the study sites are also indicated. In Nouragues two different study sites (Grand Plateau and Petit Plateau) were included in this study (see Table S1). The figure was made by Quantum GIS Geographic Information System v. 2.16 (QGIS Development Team, 2016. QGIS Geographic Information System. Open Source Geospatial Foundation Project. http://www.qgis.org/) by the authors using own data from UMR Ecofog (Kourou).

**Figure 2:**
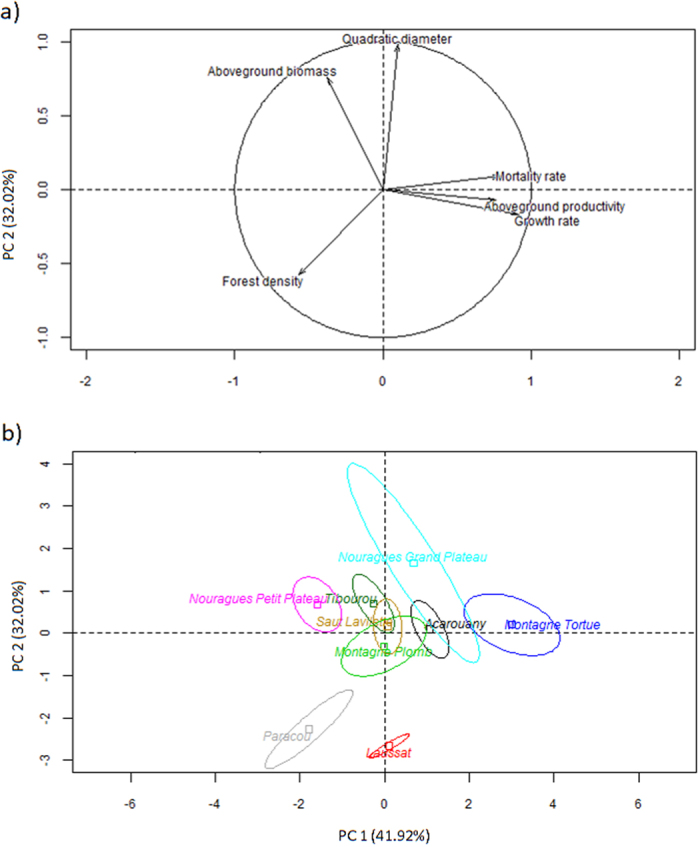
(**a**) Distribution of the study sites and (**b**) position of the variables of forest structure and forest dynamics in a Principal Component Analysis.

Quadratic diameter ranged from 240 to 296 mm, biomass from 310 to 470 t ha^−1^, and stem density from 447 to 670 stems ha^−1^ (diameter at breast height (DBH) >10 cm). Growth and mortality differed two-fold amongst sites, and productivity ranged from 3.8 to 7 t ha^−1^ y^−1^ (Table S2). Geographic distance between sites did not clearly account for the similarities or differences in forest structure and dynamics amongst sites (Figs 1 and 2). For example, quadratic diameter, productivity, and mortality were similar at distant (ca. 200 km) sites, such as Acarouany and Saut Lavillette, whereas growth and productivity were notably different at neighbouring sites, such as Nouragues-Grand Plateau and Nouragues-Petit Plateau. The first component in the PCA (PC1; 42% of the variance) was associated with forest-dynamic variability (growth, mortality and productivity), whereas the second component (PC2; 32%) was associated with variables of forest structure (biomass and quadratic diameter). Stem density contributed negatively to both components.

Total soil P was lower than in many southern and western Amazonian soils^23^; it ranged from 30 to 560 mg Kg^−1^ but many sites were below 200 mg Kg^−1^ (Table S3). Total soil N content was also lower than the content reported by Quesada *et al*.^10^ and ranged from 0.069 to 0.437%. Soil exchangeable K content ranged from 0.04 to 0.19 meq 100 g^−1^, similar to or even higher than in other tropical areas^10^,^24^. Litter P content obtained from mixed litter samples ranged from 0.01 to 0.04% of dry mass, lower than most content of litter P previously reported for tropical rainforests^25^. The litter N:P ratio ranged from 39 to 75, which also indicated a low litter P content.

### Determinants of forest structure and dynamics

Only five of the 29 predictors in this study (see Table S3 for the values of soil and litter variables) were selected in the final models (soil C:N ratio, litter K content, litter N:P ratio, total soil P content, and soil pH; Table S4). These five predictors significantly improved the null model that only included the random-site effect (see ‘Statistical analyses’ section). The remaining predictors either were not selected using the Bayesian information criterion (BIC) or were highly correlated with one of the selected predictors (Table S5). Soil C:N was highly correlated with clay (Fig. S1) and coarse-sand contents, which characterise soil texture, total soil P content was highly correlated with total soil N and K contents, and litter K content was highly correlated with litter N and P contents. All these variables were significant when analysed separately with their respective response variables, but we selected soil C:N, total soil P content, and litter K content because they slightly increased the variation explained in the model. The predictors strongly covaried with the forest-structure variables (quadratic diameter, biomass, and stem density) but not with the forest-dynamic variables (growth, mortality and productivity).

### Forest structure

**Figure 3:**
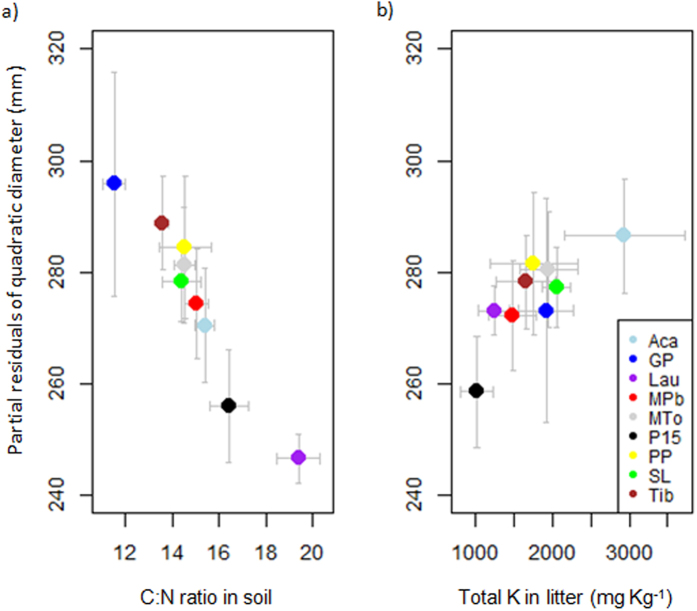
Partial residuals of (**a**) quadratic diameter vs soil C:N ratio and (**b**) quadratic diameter vs litter total K content. Abbreviations in the legend are detailed in Table S1.

**Figure 4:**
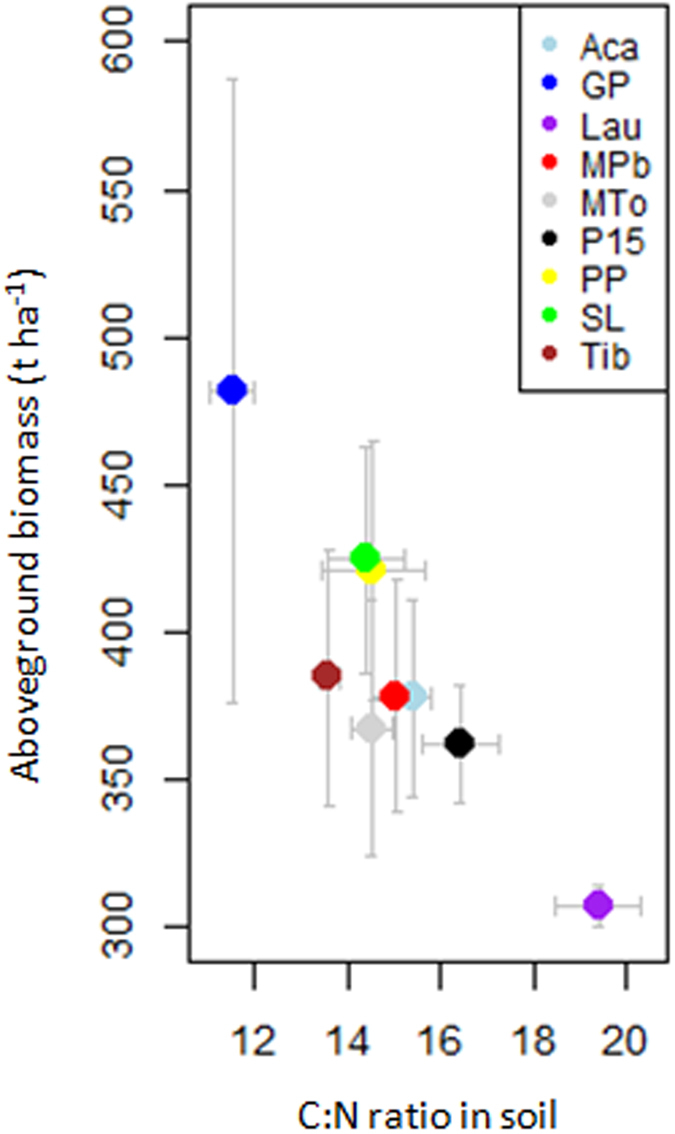
Aboveground biomass vs soil C:N ratio. Abbreviations in the legend are detailed in Table S1.

**Figure 5:**
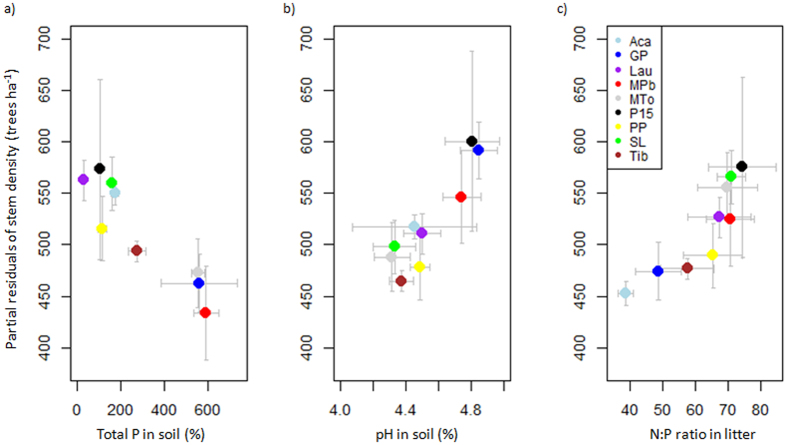
Partial residuals of (**a**) stem density vs total P content in soil, (**b**) stem density vs soil pH, and (**c**) stem density vs litter N:P ratio. Abbreviations in the legend are detailed in Table S1.

The soil C:N ratio was a robust predictor of two of the three variables of forest structure. The C:N ratio was very negatively correlated with quadratic diameter and biomass (Figs 3a and 4; Table S4). The quadratic diameter was large at the sites where soil C:N was low but was small at the sites where soil C:N was high and N content was low (Fig. 3a, Tables S2 and S3). Biomass tended to be high where soil C:N was low, whereas low biomass was associated with high soil C:N and low N content (Fig. 4, Tables S2 and S3). Total soil P content, pH, and litter N:P covaried with stem density (Fig. 5, Table S4) but were poor predictors of quadratic diameter and biomass. Litter K content was also selected as a good predictor of quadratic diameter, which covaried positively (Fig. 3b).

### Forest dynamics

None of the predictors examined in this study could directly account for the variability of growth, productivity, or mortality across sites. Growth was lowest in Nouragues-Petit Plateau (DBH increase of 0.8 mm y^−1^; Table S2), where soil P availability was amongst the lowest (Table S3). Most of the sites with higher contents of soil available P had higher growth (Fig. S2, Tables S2 and S3), but this trend was not consistent, because the site with the highest growth (DBH increase of 1.9 mm y^−1^, Montagne Tortue) had one of the lowest soil P availabilities. Montagne Tortue also had the highest mortality and a very high mean annual precipitation. Other sites with similar amounts of precipitation (Tibourou or Saut Lavillette) had much lower mortality (Tables S2 and S3), so this predictor was inconsistent despite the large gradient of precipitation across the study region. Productivity also had no clear pattern.

### Interplay between forest structure, forest dynamics, and their predictors

**Figure 6:**
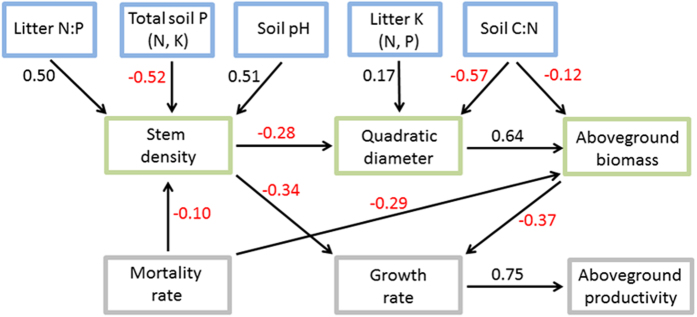
Inferred Bayesian network illustrating the relationships between the forest-structure variables (green), the forest-dynamic variables (grey), and the predictors (blue). The regression coefficients indicate a positive (black numbers) or negative (red numbers) relationship at each node; only those predictors with a statistically significant effect (Table S4) were included. Substituting litter K content with litter N or P content or substituting total soil P content with total soil N or K content (in brackets) produces nearly identical results because of their high mutual correlations (see Table S5).

Quadratic diameter was correlated positively with biomass but negatively with stem density (Figs 6 and S3, Table S6), whereas biomass and stem density were not correlated. All variables of forest dynamics, however, were positively correlated with each other; the correlations were particularly high between growth and mortality and between growth and productivity.

Biomass was negatively correlated with growth and mortality, but biomass and wood productivity were not directly associated. Stem density was negatively correlated with both growth and mortality but was not directly associated with productivity. None of the variables of forest dynamics were correlated with quadratic diameter (Figs 6 and S3, Table S6). Overall, the variables of forest structure were either negatively or not correlated with the variables of forest dynamics.

## Discussion

Our results did not fully support our hypothesis 1, that the variation in soil nutrient content amongst sites would account for the variability of forest structure and dynamics. All forest-structure variables covaried with soil or litter nutrient contents or with texture, but no clear relationships were found for the forest-dynamic variables.

Regarding forest structure variables, the soil C:N ratio was a good predictor of quadratic diameter and biomass (Figs 3a, 4 and 6; Table S4). The C:N ratio is classically used as a proxy of N availability and of the potential of nitrification^26^,^27^. The correlation in our study may indicate that N availability is limiting tree size and forest biomass, which would conflict with the hypothesis that soil P and not soil N limits tree growth in French Guiana^22^. The relationship between the soil C:N ratio and biomass (and quadratic diameter), though, does not necessarily imply a causal relationship. Moreover, none of the soil nutrient contents were clearly correlated with quadratic diameter or biomass, conflicting with hypotheses 2 and 3. This lack of correlation supports Baraloto *et al*.^28^, who argued that soil nutrients had little control over biomass across a broader gradient of Amazonian forests. Because the C:N ratio in the soil was very negatively correlated with clay content (Fig. S1), an alternative explanation could be that microbial mineralisation was less limited in clayey soils, so that the C:N ratio would be lower. This could be because a higher total P in clayey soils (Table S5) could favour a faster microbial mineralization.

The lack of correlation between soil nutrient content (e.g. total N, total P) and quadratic diameter and biomass is harder to explain. Hammond (2005) argued that long-term acidity, modest topography over much of the Guiana Shield, and isolation from the younger ranges of western, southern and central America have constrained most of the nutrients within plant biomass or retained as inaccessible stores. The low dependence of biomass on direct nutrient uptake from poor soils may be due to alternative mechanisms such as nutrient cycling from litter or nutrient resorption during leaf senescence. Litter nutrient contents (total K, total N, and total P) were positively correlated with quadratic diameter (Figs 3b and 6, Table S4), suggesting that nutrient uptake from litter was also an important mechanism to reduce the dependence on soil nutrients in nutrient-poor forests^20^,^21^. Our results suggest that the content of nutrients in the soil in nutrient-poor soils is not a good proxy of the flux absorbed by roots, not even for the most limiting nutrients such as P. We therefore hypothesise that the amount of P in soil is much lower than the annual P flux by resorption or by litterfall and decomposition. Sardans and Peñuelas^29^ argued that trees growing in soils with low P availabilities may have developed long-term adaptive mechanisms to store P in biomass, mainly in wood, thereby slowing the loss of P from the ecosystem, and predicted an increase in the P:N ratio in biomass with aging. This is in agreement with Heineman *et al*.^30^, who suggested that tropical trees may be under selection to allocate excess P to storage to mitigate P limitation when the P demands of plant growth exceed P supply from the soil. Sardans and Peñuelas^29^ also argued that this trend to store more P than N with increasing biomass should be more accentuated in slow-growing, large, long-lived species in mature forests. Biomass storage may account for the accumulation of P in biomass during secondary succession in Amazonian forests even though soil P availability decreases^31^. Nutrient resorption and the storage of large amounts of limiting nutrients in forests with high biomass may thus be a plausible mechanism to counterbalance the low availability of nutrients in the soil^29^ and to reduce the dependence on direct nutrient uptake. The accumulation of biomass (310–469 t ha^−1^) was generally much larger than in more nutrient-rich Amazonian sites (generally below 300 t ha^−1^; ref. 10), suggesting that the potential for the aboveground storage of nutrients is also large. Future studies on nutrient budgets in forest compartments are needed to confirm or reject this hypothesis.

Sites with low soil total N and total P contents and high litter N:P ratios had very high stem densities, small quadratic diameters, and low biomasses (Figs 2, 5a,b and 6; Tables S2 and S3). In contrast, sites with comparatively higher soil total N and total P contents and lower litter N:P ratios had low stem densities and large quadratic diameters. This is consistent with Paoli *et al*.^32^, who found that stem density in Borneo was negatively correlated with soil nutrient content, and with Baraloto *et al*.^28^, who showed that the density of small stems in Amazonian forests was higher in nutrient-poor soils. We hypothesise that growth is less limited by light at sites limited by both P and N than at nutrient-rich sites, because of the lower accumulation of biomass and lower overall leaf area index. Previous studies found that light availability in the understory may increase with decreasing availability of soil resources^33^. The higher availability of light than at nutrient-rich sites may enhance the germination and recruitment of trees, leading to high stem density. Soil pH in our study was positively correlated with stem density, but the low overall pH and the small variation in pH amongst our study sites (4.3–4.8, Table S3) would unlikely cause any change in stem density, so this correlation may not be attributable to any known mechanism or imply a causal relationship.

With regard to forest dynamics variables, our results do not support our hypothesis 2, which predicted that higher nutrient content would enhance productivity. Some studies have provided evidence that productivity is accelerated with increasing soil nutrient content across large tropical regions (e.g. ref. 7), but we did not find such a relationship with either soil or litter nutrient content, even though growth and productivity differed two-fold amongst sites. Contrary to hypothesis 2 and also to hypothesis 3 (that P content would be the most important predictor), the three- and six-fold differences in available P and total N contents, respectively, did not suggest any consistent relationships with the variability of growth and productivity amongst sites. This may be due to different non-exclusive reasons. Firstly, the range of variability of soil P availability amongst sites may be too narrow to detect differences, which is unlikely because the sensitivity to small changes in soil nutrient contents may increase in regions strongly limited by nutrients. Moreover, Baribault *et al*.^15^ showed that similarly narrow changes in soil P availability (1.99–5.08 mg Kg^−1^) were enough to detect changes in basal-area increase in species with low wood densities in a P-limited forest. Secondly, detecting relationships with soil nutrient contents in highly diverse forests is difficult; however, we assume it should be possible if the relationships existed, as demonstrated by other studies of highly diverse forests^9^,^10^. Thirdly, growth and productivity in nutrient-limited soils may respond to alternative nutrient-cycling mechanisms other than the direct absorption of nutrients from the soil^34^, such as the uptake of nutrients from litter by mycorrhizae^21^ or the resorption of nutrients during leaf senescence^35^.

Litter N:P was generally very high, so litter decomposition and the release of nutrients should be slow^13^,^35^. Therefore, we hypothesise that the capacity of nutrient cycling by resorption at our study sites may influence growth and productivity more strongly than nutrient cycling by litter decomposition. This is in agreement with Hammond (2005), who argued that nutrient resorption during leaf senescence regulates the mass balance of nutrients and carbon fluxes in most forests of the Guiana Shield. The capacity of biomass to store nutrients and the reduction of nutrient release from the biomass to the soil, rather than the content of nutrients in the soil, may strongly control the growth and productivity on these nutrient-limited soils. Nutrient-use efficiency is higher in low-nutrient than in high-nutrient environments^35^, and P:N resorption ratios are generally higher in P-limited soils^29^. Some studies have shown that resorption of the more limiting nutrients is common in nutrient-poor soils^36^. Lovelock *et al*.^37^ argued that low P availability exerted a strong selective pressure, favouring populations that were more efficient at resorbing P in nutrient-limited tropical forests. Heineman *et al*.^30^ reported that P allocation may be a key component of specialised edaphic niches in tropical regions worldwide and that the allocation of limiting nutrients to woody biomass is an important functional characteristic influencing species distributions along edaphic gradients in tropical forests. Hättenschwiler *et al*.^38^ suggested that the degree of nutrient limitation vary amongst species in the same forest. We therefore predict that the species composition at a given site and the species-specific nutrient-use efficiencies will ultimately control site-level growth and productivity, together with other abiotic regulators such as water availability^14^,^39^.

Mortality was not correlated with any of the soil characteristics analysed. Positive relationships between soil nutrient content and mortality have been described for tree seedlings in French Guiana^40^ and for old-growth stands across Amazonia^41^. In both cases, this positive relationship was interpreted as an indirect effect: the high nutrient content would accelerate seedling growth^40^ or coarse-wood productivity across Amazonia^7^ and therefore increase the competition for resources (light or water) amongst trees. Other factors may have direct effects on mortality, such as exceptionally heavy rains and waterlogging that increase soil instability^42^. This higher mortality leads to a lower basal area and to a higher proportion of light-demanding species and to a faster increase in tree diameter but to a lower productivity^43^. Our data indicated a positive correlation between productivity and mortality (Fig. S3, Table S6), suggesting that the variation in mortality was mainly driven by variation in productivity and thus by differences in competition for resources and not by a direct environmental factor of mortality. Conversely, mortality may act as a natural clearing that reduces competition and enhances productivity.

Concerning the interplay between forest structure and forest dynamics variables, our results suggest that growth is strongly influenced by changes in stem density and biomass, which are in turn controlled by soil chemical and physical characteristics (Fig. 6). Growth is therefore only indirectly influenced by soil characteristics. Changes in stem density and biomass were also strongly influenced by mortality, suggesting that the variables of forest structure and dynamics are markedly mutually dependent. The negative correlation between tree density and quadratic diameter (Figs 6 and S3, Table S6) was likely due to the self-thinning law^44^, which states that a high stem density and a high quadratic diameter cannot co-occur because of resource competition. We hypothesise that large trees with high biomass accumulation and high potential to store nutrients^29^ at nutrient-poor sites may outcompete small trees with low biomass accumulation and lower nutrient-storage potential (although small trees can also be outcompeted because they have smaller root systems and less potential to obtain water in dry seasons, and absorb less light and thus produce fewer photosynthates to allocate to their mycorrhizal symbionts). Competition may also account for the negative correlation between stem density and growth. The importance of stem density is also apparent in Fig. 2, which shows that stem density was correlated negatively with forest-dynamic variables (first axis in the PCA) and also with the other forest-structure variables analysed (second axis in the PCA). Stem density is thus key to understanding the interplay between forest structure and dynamics.

The positive correlation between mortality and growth and the negative correlation between biomass and mortality and growth (Fig. S3, Table S6) support the self-maintaining positive feedback mechanism of forest dynamics proposed by Quesada *et al*.^10^ for Amazonian forests. These authors suggested that under low disturbance, mortality would decrease and therefore the loss of biomass or the reduction of stem density due to gap formation would also decrease, which would lower the light levels inside the forests and also the growth. We therefore hypothesise that ecosystem-level strategies and adaptations to low soil nutrient contents, low levels of disturbance, and long-term stability in French Guiana^45^,^46^ account for the rather to low productivity (3.8–7 t ha^−1^ y^−1^) and the high biomass (310–469 t ha^−1^) at our study sites (and possibly in other undisturbed nutrient-poor forests in the Amazon Basin), compared to other nutrient-richer and more disturbed Amazonian forests^9^,^10^.

## Materials and Methods

### Study sites and data collection

French Guiana is located on the Guiana Shield (northeastern Amazonia). This South American region hosts substantial geodiversity^47^ and a wide variety of soils^48^. The data were collected at nine study sites in natural, unlogged primary forests across the coastal zone (Fig. 1, Table S1).

The study sites belonged to the Guyafor network (www.ecofog.gf/spip.php?article364), which was designed to assess the spatiotemporal variations in forest structure, composition, and dynamics. The network consists of several permanent plots that are monitored over time, with varying census frequencies amongst sites (Table S1). Each site consists of several contiguous 1-ha plots (except for 1.56-ha plots at Paracou); each plot was used as a replicate within each site in the statistical analyses. All trees ≥10 cm DBH were measured in each census every 2–5 years, and more than two-thirds of the stems were identified with high levels of certainty by our botanical team using vouchers deposited at the regional herbarium in Cayenne. All living and dead trees were mapped, tagged, and inventoried in the database. A total of 34 134 monitored trees have been considered in the analyses.

Soil and litter samples were collected between 2008 and 2010 at all study sites. Each soil sample was a composite of 10 mixed soil cores to a depth of 20 cm collected randomly within a 40 × 10 m rectangle. Five rectangles were sampled across each site and thus a total of five composite soil samples were obtained per site. All rectangles were sampled on the dominant topography of each site (either a plateau or a slope; Table S1). Litter samples were also collected from each of the five rectangles and consisted of 10 dead leaves from the top layer around each of the 10 cores, with no more than two leaves from the same species. These leaves showed no apparent signs of decomposition, so we assumed that nutrient loss from the litter was minimal before collection. Tree diversity at our study sites was exceptionally high (150–200 tree species ha^−1^). The litter of a single tree may locally dominate its immediate surroundings, so we assumed that collecting litter from different species better represented the mixture of litter on the ground at the site level.

Soil samples were analysed for: carbon (C), nitrogen (N), total phosphorus (total P), available phosphorus (available P, extracted by the Olsen method), exchangeable calcium (Ca), magnesium (Mg), potassium (K), sodium (Na), aluminium (Al), and magnesium (Mg) contents, cation exchange capacity (CEC, cobaltihexamine method at soil pH), soil texture (% of clay, fine silt, coarse silt, fine sand, and coarse sand), and pH in water. Litter samples were analysed for: C, N, P, Ca, Mg, K, Na, and ash contents. We also included mean annual precipitation (source: Méteo France; see ref. 49 for more details) in our analyses, because precipitation was highly variable across the study sites (Fig. 1, Table S1). We assigned the same mean site values of soil, litter, and precipitation variables to all 1-ha plots (1.56-ha plots at Paracou) within each site. See Table S3 for the list of measured variables in soil and litter and see ref. 49 for a complete characterisation of the soil profiles at the study sites and for precipitation data.

### Estimation of the variables of forest structure and forest dynamics

We characterised forest structure by estimating the quadratic diameter, stem density, and biomass in each of the plots at each site. The quadratic diameter was defined as the quadratic mean tree size in a plot^50^:


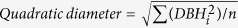


where *DBH*_*i*_ is the mean diameter at breast height (mm) of the *i*^*th*^ tree for a given census period, and *n* is the number of trees in the plot. Stem density was estimated by counting the number of living trees with DBH > 10 cm in each plot (trees ha^−1^). The biomass of each tree was estimated using a pantropical allometric equation^51^:


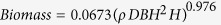


where *ρ* is wood density (g cm^−3^), *DBH* is diameter at breast height (cm), and *H* is tree height (m). Data for wood density were obtained from the global wood-density database^52^,^53^ for each species (58.9% of the trees). We assigned the mean genus value (11.3%) to incompletely identified species and the plot-level mean (29.7%) in cases of no information or uncertain identity. *H* was estimated using an accurate regionally developed and validated height model based on forest structure (details in ref. 54. Total dry biomass (t ha^−1^) was calculated by summing the biomasses of all living trees in a plot.

Forest dynamics was assessed by growth rate, mortality rate, and productivity. Growth rate (growth hereafter) was calculated as the mean increase in DBH (mm y^−1^) in each plot, and mortality rate (λ_m_, mortality hereafter) was estimated using estimators of instantaneous mortality^55^ (equations 1 and 2):


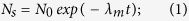



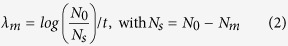


where *N*_*s*_ is the number of survivors per plot, *N*_*0*_ is the initial number of trees, *N*_*m*_ is the number of dead trees per plot, and *t* is the time between two consecutive censuses. Productivity was calculated as the increase in total aboveground wood biomass in each plot (t ha^−1^ y^−1^) including all the living trees between two censuses.

To account for the different time periods between censuses over time and sites (see Table S1), we first calculated the variables of forest structure and dynamics for each period between two consecutive censuses and then calculated a weighted mean (weighted by the time between censuses) for each plot at each site (Table S2).

### Statistical analyses

We first conducted a principal component analysis (PCA) using the ‘FactoMineR’ package^56^ as implemented in R 3.2^57^ to identify the relationships between variables of forest structure and dynamics amongst sites. All variables were then analysed using linear mixed models with the ‘lme4’ package^58^. We first designed a null model that only included the random-site effect, which accounted for the spatial dependencies in our sampling design (several plots per site). The initial screening was based on univariate analyses to identify the potential predictors, and we then built a full model, where all potential predictors were added to the null model. We only used the predictors that were poorly correlated with each other to avoid problems of collinearity (Table S5). Only a few of the soil and litter variables analysed were thus retained. We used an automated model selection based on the BIC to identify the best models using the ‘MuMin’ package^59^. The BIC values were always much lower for the best model than for the second-best models (∆BIC > 2), so we did not perform model averaging and directly reported the best model estimates. The *p*-values of the best model were calculated with the ‘nlme’ package^60^. Conditional *R*^2^ was estimated following the method described in ref. 61. If more than one predictor was selected in the final model, we calculated the partial residuals of each predictor with the ‘effects’ package^62^ to determine the effect of each predictor on a given variable by excluding the effects of the other predictors.

We used the predictors that became significant in the mixed-model analyses to infer a flow diagram, converted into a directed acyclic graph (DAG) using Bayesian networks with the ‘abn’ package^63^, which allows accounting for dependencies amongst plots at each site. The significant predictor variables from the mixed models were used as external, unidirectional parents at the nodes. In this DAG, we imposed the direction of the arrows from the predictor variables to the response variables and also between the response variables. We assumed that the DAG chosen was not necessarily the best diagram for maximising the likelihood, but our aim was to test the strength of the relationships in a single logical framework. The correlations between the modelled variables were also estimated by Pearson’s product moment correlations.

## Additional Information

**How to cite this article:** Grau, O. *et al*. Nutrient-cycling mechanisms other than the direct absorption from soil may control forest structure and dynamics in poor Amazonian soils. *Sci. Rep.* **7**, 45017; doi: 10.1038/srep45017 (2017).

**Publisher's note:** Springer Nature remains neutral with regard to jurisdictional claims in published maps and institutional affiliations.

## Supplementary Material

Supplementary Information
