# Supplementary material for: Nutrient-cycling mechanisms other than the direct absorption from soil may control forest structure and dynamics in poor Amazonian soils
Source: Sci Rep. 2017 Mar 23;7:45017. doi: 10.1038/srep45017 (PMC5362906; doi:10.1038/srep45017)
Supplement: Supplementary Information [file srep45017-s1.pdf]

SUPLEMENTARY INFORMATION OF THE ARTICLE: "NUTRIENT-CYCLING MECHANISMS OTHER THAN THE DIRECT ABSORPTION FROM SOIL MAY CONTROL FOREST STRUCTURE AND DYNAMICS IN POOR AMAZONIAN SOILS"

Oriol Grau, Josep Peñuelas, Bruno Ferry, Vincent Freycon, Lilian Blanc, Mathilde Desprez, Christopher Baraloto, Jérome Chave, Laurent Descroix, Aurélie Dourdain, Stephan Guitet, Ivan A. Janssens, Jordi Sardans, and Bruno Hérault

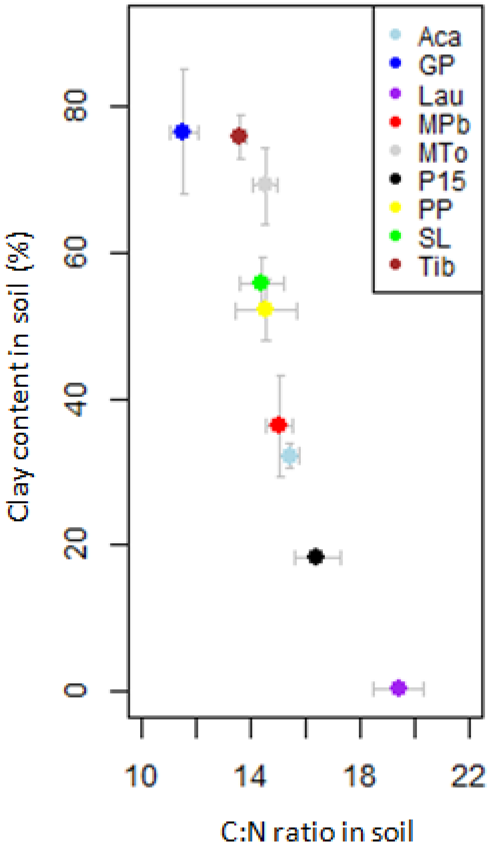

Figure S1. Clay content vs soil C:N ratio. Abbreviations in the legend are detailed in Table S1.

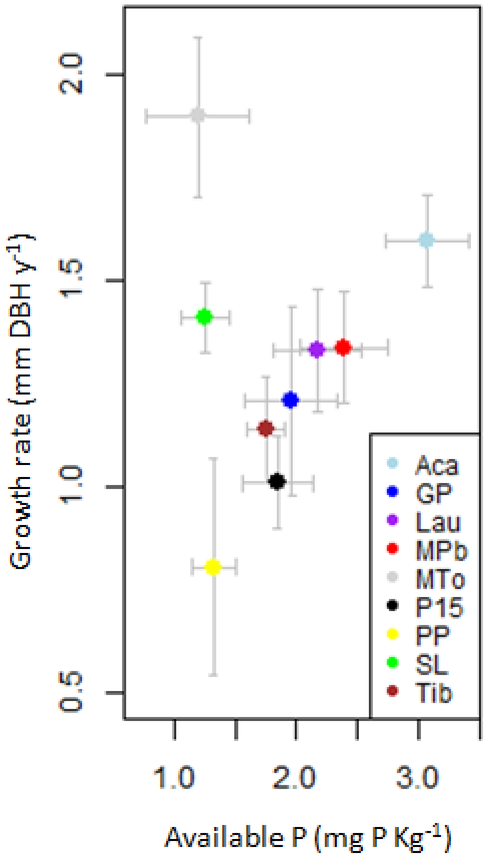

Figure S2. Growth rate vs soil available P content. Abbreviations in the legend are detailed in Table S1.

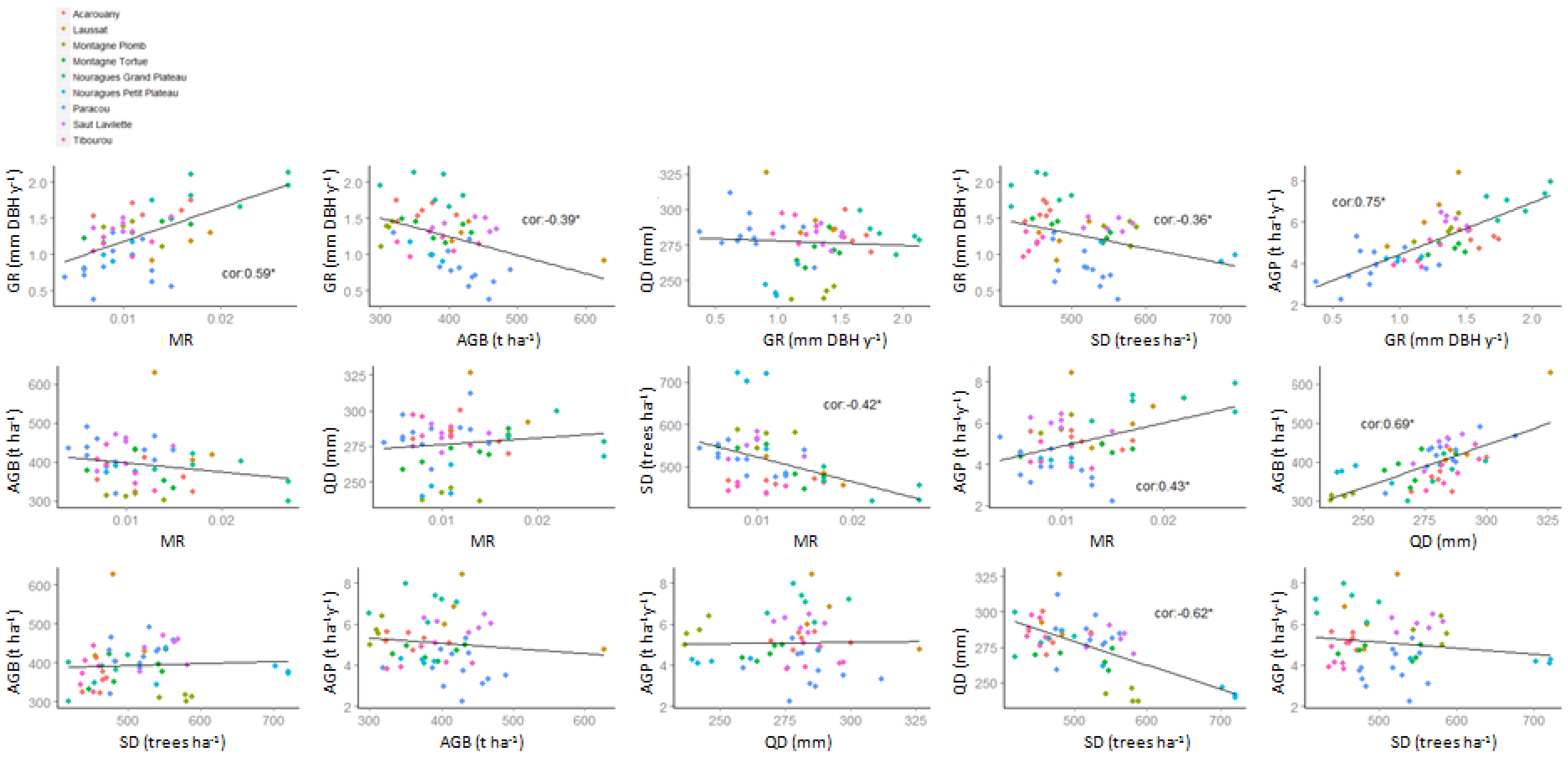

Figure S3. Correlations amongst forest-structure and forest-dynamic variables. 'cor' indicates the significant Pearson's product moment correlations (*); AGB, Aboveground biomass; AGP, Aboveground woody productivity; GR, growth rate; MR, mortality rate; QD, quadratic diameter; SD, stem diameter.

Table S1. Characteristics of the study sites. The abbreviations of the study sites in the figures are also indicated.

Site	Number of monitored trees	Number of 1-ha plots	Latitude (°)	Longitude (°)	Elevation (m)	Starting year	Number of censuses	Reference topography	Geological substrate	Soil type	Mean annual precipitation (mm)
Acarouany (Aca)	2957	6	5.543146	-53.812982	49	2006	4	gentle slope (3-10%)	Migmatites Caraibe	Ferralsol	2800
Laussat (Lau)	2387	4	5.479167	-53.596433	57	2009	2	plateau	White sand	Arenosol	2800
Montagne Plomb (MPb)	3301	6	5.006467	-52.946968	114	2005	4	moderate slope (8-23%)	Paramaca	Ferralsol and Plinthosol	3380
Montagne Tortue (MTo)	3401	6	4.217797	-52.41255	160	2002	5	moderate slope (10-16%)	Paramaca	Ferralsol	3930
Nouragues-Grand Plateau (GP)	3069	4	4.085704	-52.676461	125	2000**	3	moderate slope (10-20%)	Paramaca	Ferralsol	2960
Nouragues-Petit Plateau (PP)	7576	12	4.082899	-52.683623	128	2000**	3	moderate slope (5-20%)	Migmatites Caraibe	Ferralsol	2960
Paracou (P15)	5036	4*	5.277326	-52.927279	40	2001**	11	moderate slope (2-32%)	Schist (Bonidoro)	Acrisol	2821
Saut Lavillette (SL)	3589	6	4.148613	-52.20247	84	2003	3	plateau	Migmatites Caraibe	Ferralsol	3930
Tibourou (Tib)	2818	6	4.398647	-52.277666	77	2006	3	gentle slope (1-12%)	Schist (Bonidoro)	Ferralsol	3830

*Each plot at Paracou had an area of 1.56 ha. **These plots had been monitored previously, but only the data collected after the year 2000 were used in order to standardise the census periods and methods of data collection amongst sites.

Table S2. Site-level means (±sd) of quadratic diameter, aboveground biomass, stem density, growth rate, mortality rate, and aboveground wood productivity.

Site	Period	Plot area (ha)	Quadratic diameter (mm)	Aboveground biomass (t ha^{-1})	Stem density (trees ha^{-1})	Growth rate (mm DBH increase year^{-1})	Mortality rate (λ_m)	Aboveground wood productivity (t ha^{-1}y^{-1})
Acarouany	2006-2013	1	283±10	358±33	460±12	1.597±0.11	0.0123±0.004	5.2±0.34
Laussat	2009-2013	1	240±4	310±7	572±20	1.331±0.15	0.0108±0.003	5.6±0.58
Montagne Plomb	2005-2013	1	271±10	385±40	508±46	1.338±0.13	0.0118±0.004	4.622±0.32
Montagne Tortue	2002-2013	1	283±10	374±43	457±33	1.899±0.19	0.0205±0.006	7.020±0.66
Nouragues (Grand Plateau)	2000-2008	1	297±20	470±106	486±28	1.210±0.22	0.0150±0.004	6.493±1.52
Nouragues (Petit Plateau)	2000-2008	1	284±13	423±44	516±31	0.806±0.26	0.0090±0.003	3.814±0.86
Paracou (P15)	2001-2014	1.56	247±10	371±20	671±88	1.011±0.11	0.0098±0.006	4.210±0.10
Saut Lavillette	2003-2012	0.99	281±7	432±38	550±26	1.411±0.08	0.0102±0.003	6.040±0.32
Tibourou	2006-2012	1	287±8	380±43	447±10	1.140±0.13	0.0096±0.002	4.317±0.54

Table S3. Site-level means of soil and litter chemical characteristics.

	Soil																		Litter										
Site	Total C (%)	Total N (%)	Available P (mg Kg^{-1})	Total P (mg Kg^{-1})	pH (in water)	Clay (%)	Fine silt (%)	Coarse silt (%)	Fine sand (%)	Coarse sand (%)	CEC (meq 100 g^{-1})	Ca (meq 100 g^{-1})	Mg (meq 100 g^{-1})	K (meq 100 g^{-1})	Na (meq 100 g^{-1})	Al (meq 100 g^{-1})	Mn (meq 100 g^{-1})	C:N	N:available P	Total C (%)	Total N (%)	Total P (mg Kg^{-1})	Ash ((mg Kg^{-1})	Total Ca ((mg Kg^{-1})	Mg (mg Kg^{-1})	K (mg Kg^{-1})	Na (mg Kg^{-1})	C:N	N:P
Acarouany	2.10	0.14	3.08	171.60	4.45	32.18	2.12	0.90	10.80	53.96	2.04	0.35	0.19	0.06	0.14	0.98	0.00	15.39	451	48.61	1.85	478	6566	6.596	2.910	2.938	1.518	26.35	38.78
Laussat	1.34	0.07	2.17	30.60	4.50	0.44	0.42	0.62	3.48	95.06	1.23	0.41	0.22	0.04	0.05	0.02	0.01	19.40	331	51.13	1.07	162	5476	13.796	2.534	1.240	616	47.89	67.52
Montagne Plomb	6.43	0.43	2.39	592.20	4.74	36.34	27.34	3.60	9.46	23.30	5.39	0.91	0.45	0.19	0.14	2.00	0.02	15.05	1816	51.74	1.15	164	4396	13000	2.004	1.482	846	45.20	70.88
Montagne Tortue	3.82	0.26	1.19	553.6	4.31	69.16	9.30	3.96	4.50	13.14	2.59	0.22	0.23	0.09	0.12	1.56	0.03	14.52	2471	51.54	1.56	228	6448	10312	2.360	1.950	954	32.99	69.87
Nouragues-Grand Plateau	2.71	0.23	1.96	560.60	4.85	76.42	8.42	2.14	3.00	10.04	3.10	0.61	0.41	0.08	0.07	0.60	0.34	11.54	1230	49.84	1.55	322	8056	13644	2.766	1.916	722	32.20	48.74
Nouragues-Petit Plateau	2.33	0.16	1.32	114.00	4.49	52.06	3.02	1.50	7.28	36.12	2.75	0.09	0.18	0.06	0.07	1.56	0.01	14.55	1227	51.04	1.26	196	5088	6458	2.248	1.752	978	40.40	65.52
Paracou (P15)	2.10	0.13	1.85	104.00	4.81	18.35	4.35	2.88	27.87	46.58	1.12	0.21	0.26	0.08	0.11	0.43	0.00	16.42	701	50.82	1.31	178.6	8760	5950	2.764	1.010.2	595	39.07	74.59
Saut Lavillette	3.07	0.21	1.25	158.40	4.33	55.78	14.10	1.22	4.12	24.84	2.33	0.12	0.18	0.07	0.09	1.56	0.01	14.39	1743	53.48	1.34	188	4252	6326	2.324	2.052	1.320	40.16	71.18
Tibourou	4.25	0.31	1.75	272.80	4.38	75.82	6.64	2.62	6.44	8.50	3.13	0.25	0.27	0.09	0.09	1.74	0.01	13.61	1786	51.45	1.61	282	4180	8344	2.550	1.654	794	32.33	57.68

Table S4. Selected predictors in the mixed models. The significant predictors are in bold type.

Variable	R^2 (fixed factors)	Selected predictors	Estimates	p
**Quadratic diameter**	0.63	Intercept	277.137	
		**Soil C:N**	**-12.13**	**0.0003 (***)**
		**Litter K content**[a]	**5.33**	**0.0175 (*)**
**Aboveground biomass**	0.89	Intercept	392.427	
		**Soil C:N**	**-31.97**	**0.0039 (**)**
**Stem density**	0.69	Intercept	514.658	
		**Litter N:P**	**35.43**	**0.0041 (**)**
		**Total soil P**[b]	**-41.50**	**0.0026 (**)**
		**Soil pH**	**37.54**	**0.0034 (**)**
**Growth rate**	-	None	-	-
**Mortality rate**	-	None	-	-
**Aboveground wood productivity**	-	None	-	-

[a]Litter N and P contents were also significant predictors but were highly correlated with litter K content, so only one was included in the final models; we chose litter K content because it increased model robustness. [b]Total N and K contents in the soil were also significant predictors but were highly correlated with soil total P content, so only one was included in the final models; we chose soil total P content because it increased model robustness. No significant predictors were selected for growth rate, mortality rate, or aboveground wood productivity. ***, $p < 0.0005$; **, $p < 0.005$; *, $p < 0.05$.

Table S5. Pearson's product moment correlation coefficients amongst soil and litter chemical characteristics and mean annual precipitation (MAP).

		Soil																			Litter										
		Total C (%)	Total N (%)	Available P (mg Kg^{-1})	Total P (mg Kg^{-1})	pH (in water)	Clay (%)	Fine silt (%)	Coarse silt (%)	Fine sand (%)	Coarse sand (%)	CEC (meq 100 g^{-1})	Ca (meq 100 g^{-1})	Mg (meq 100 g^{-1})	K (meq 100 g^{-1})	Na (meq 100 g^{-1})	Al ((meq 100 g^{-1})	Mn (meq 100 g^{-1})	C:N ratio	N:available P ratio	Total C (mg Kg^{-1})	Total N ((mg Kg^{-1})	Total P ((mg Kg^{-1})	Ash (mg Kg^{-1})	Total Ca (mg Kg^{-1})	Mg (mg Kg^{-1})	K (mg Kg^{-1})	Na (mg Kg^{-1})	C:N ratio	N:P ratio	MAP (mm)
Soil	**Total C (%)**	1.00	0.98	-0.03	0.72	0.05	0.38	0.89	0.71	-0.12	-0.62	0.91	0.56	0.63	0.94	0.53	0.79	-0.05	-0.37	0.72	0.37	-0.06	-0.20	-0.47	0.26	-0.66	-0.07	-0.02	0.09	0.23	0.58
	**Total N (%)**	0.98	1.00	-0.07	0.79	0.08	0.54	0.86	0.71	-0.20	-0.74	0.92	0.55	0.68	0.90	0.44	0.78	0.12	-0.53	0.76	0.33	0.03	-0.12	-0.42	0.28	-0.59	-0.02	-0.03	-0.02	0.12	0.60
	**Available P (mg Kg^{-1})**	-0.03	-0.07	1.00	-0.03	0.34	-0.47	-0.03	-0.27	0.19	0.42	0.07	0.56	0.25	0.13	0.35	-0.29	0.02	0.25	-0.61	-0.70	0.25	0.58	0.16	0.20	0.43	0.32	0.19	-0.19	-0.61	-0.58
	**Total P (mg Kg^{-1})**	0.72	0.79	-0.03	1.00	0.26	0.57	0.66	0.73	-0.29	-0.70	0.74	0.63	0.76	0.71	0.37	0.44	0.53	-0.61	0.67	0.00	0.20	0.07	0.05	0.54	-0.30	0.11	-0.11	-0.22	-0.07	0.38
	**pH (in water)**	0.05	0.08	0.34	0.26	1.00	-0.21	0.21	0.20	0.46	0.01	0.19	0.60	0.73	0.33	-0.02	-0.38	0.54	-0.12	-0.34	-0.35	-0.26	-0.09	0.59	0.35	0.18	-0.41	-0.53	0.18	-0.02	-0.60
	**Clay (%)**	0.38	0.54	-0.47	0.57	-0.21	1.00	0.21	0.38	-0.44	-0.91	0.42	-0.11	0.21	0.16	0.00	0.57	0.46	-0.91	0.73	0.14	0.51	0.25	-0.14	-0.04	-0.11	0.34	0.16	-0.55	-0.24	0.62
	**Fine silt (%)**	0.89	0.86	-0.03	0.66	0.21	0.21	1.00	0.56	-0.10	-0.49	0.84	0.63	0.64	0.91	0.45	0.64	0.04	-0.30	0.60	0.48	-0.26	-0.32	-0.38	0.30	-0.69	-0.09	0.04	0.29	0.34	0.47
	**Coarse silt (%)**	0.71	0.71	-0.27	0.73	0.20	0.38	0.56	1.00	0.23	-0.63	0.51	0.29	0.54	0.71	0.54	0.47	0.06	-0.33	0.71	0.20	0.03	-0.26	0.12	0.17	-0.36	-0.30	-0.35	-0.07	0.39	0.46
	**Fine sand (%)**	-0.12	-0.20	0.19	-0.29	0.46	-0.44	-0.10	0.23	1.00	0.16	-0.30	-0.12	-0.01	0.06	0.42	-0.24	-0.31	0.25	-0.35	-0.20	-0.05	-0.08	0.54	-0.48	0.31	-0.35	-0.21	0.01	0.24	-0.38
	**Coarse sand (%)**	-0.62	-0.74	0.42	-0.70	0.01	-0.91	-0.49	-0.63	0.16	1.00	-0.59	-0.07	-0.41	-0.47	-0.27	-0.69	-0.37	0.90	-0.81	-0.23	-0.39	-0.10	0.09	0.08	0.24	-0.18	-0.09	0.45	0.04	-0.65
	**CEC (meq 100 g^{-1})**	0.91	0.92	0.07	0.74	0.19	0.42	0.84	0.51	-0.30	-0.59	1.00	0.68	0.71	0.88	0.33	0.74	0.18	-0.49	0.58	0.18	-0.07	-0.07	-0.46	0.37	-0.64	0.06	0.03	0.08	0.00	0.35
	**Ca (meq 100 g^{-1})**	0.56	0.55	0.56	0.63	0.60	-0.11	0.63	0.29	-0.12	-0.07	0.68	1.00	0.88	0.72	0.24	0.04	0.39	-0.08	0.01	-0.20	-0.18	0.02	-0.01	0.75	-0.20	-0.10	-0.25	0.20	-0.14	-0.18
	**Mg (meq 100 g^{-1})**	0.63	0.68	0.25	0.76	0.73	0.21	0.64	0.54	-0.01	-0.41	0.71	0.88	1.00	0.76	0.15	0.12	0.59	-0.39	0.23	-0.10	-0.14	-0.08	0.14	0.66	-0.19	-0.27	-0.46	0.10	-0.04	-0.04
	**K (meq 100 g^{-1})**	0.94	0.90	0.13	0.71	0.33	0.16	0.91	0.71	0.06	-0.47	0.88	0.72	0.76	1.00	0.55	0.61	0.01	-0.25	0.53	0.25	-0.20	-0.25	-0.28	0.34	-0.60	-0.19	-0.14	0.21	0.26	0.32
	**Na (meq 100 g^{-1})**	0.53	0.44	0.35	0.37	-0.02	0.00	0.45	0.54	0.42	-0.27	0.33	0.24	0.15	0.55	1.00	0.44	-0.35	-0.08	0.28	-0.14	0.40	0.29	0.06	-0.26	-0.05	0.32	0.40	-0.35	-0.05	0.22
	**Al (meq 100 g^{-1})**	0.79	0.78	-0.29	0.44	-0.38	0.57	0.64	0.47	-0.24	-0.69	0.74	0.04	0.12	0.61	0.44	1.00	-0.27	-0.48	0.79	0.44	0.13	-0.07	-0.66	-0.22	-0.68	0.24	0.38	-0.11	0.14	0.71
	**Mn (meq 100 g^{-1})**	-0.05	0.12	0.02	0.53	0.54	0.46	0.04	0.06	-0.31	-0.37	0.18	0.39	0.59	0.01	-0.35	-0.27	1.00	-0.62	0.03	-0.32	0.20	0.26	0.46	0.52	0.30	0.09	-0.26	-0.27	-0.41	-0.18
	**C:N ratio**	-0.37	-0.53	0.25	-0.61	-0.12	-0.91	-0.30	-0.33	0.25	0.90	-0.49	-0.08	-0.39	-0.25	-0.08	-0.48	-0.62	1.00	-0.55	0.04	-0.52	-0.36	-0.06	0.05	-0.01	-0.38	-0.19	0.58	0.36	-0.37
	**N:available P ratio**	0.72	0.76	-0.61	0.67	-0.34	0.73	0.60	0.71	-0.35	-0.81	0.58	0.01	0.23	0.53	0.28	0.79	0.03	-0.55	1.00	0.58	0.09	-0.25	-0.38	0.06	-0.61	0.03	0.06	-0.10	0.34	0.89
Litter	**Total C (mg Kg^{-1})**	0.37	0.33	-0.70	0.00	-0.35	0.14	0.48	0.20	-0.20	-0.23	0.18	-0.20	-0.10	0.25	-0.14	0.44	-0.32	0.04	0.58	1.00	-0.54	-0.76	-0.58	-0.06	-0.71	-0.40	-0.08	0.56	0.78	0.71
	**Total N (mg Kg^{-1})**	-0.06	0.03	0.25	0.20	-0.26	0.51	-0.26	0.03	-0.05	-0.39	-0.07	-0.18	-0.14	-0.20	0.40	0.13	0.20	-0.52	0.09	-0.54	1.00	0.90	0.24	-0.32	0.60	0.78	0.54	-0.99	-0.77	0.14
	**Total P (mg Kg^{-1})**	-0.20	-0.12	0.58	0.07	-0.09	0.25	-0.32	-0.26	-0.08	-0.10	-0.07	0.02	-0.08	-0.25	0.29	-0.07	0.26	-0.36	-0.25	-0.76	0.90	1.00	0.25	-0.18	0.68	0.83	0.57	-0.87	-0.95	-0.21
	**Ash (mg Kg^{-1})**	-0.47	-0.42	0.16	0.05	0.59	-0.14	-0.38	0.12	0.54	0.09	-0.46	-0.01	0.14	-0.28	0.06	-0.66	0.46	-0.06	-0.38	-0.58	0.24	0.25	1.00	0.00	0.69	-0.08	-0.28	-0.33	-0.19	-0.54
	**Total Ca (mg Kg^{-1})**	0.26	0.28	0.20	0.54	0.35	-0.04	0.30	0.17	-0.48	0.08	0.37	0.75	0.66	0.34	-0.26	-0.22	0.52	0.05	0.06	-0.06	-0.32	-0.18	0.00	1.00	-0.19	-0.26	-0.50	0.33	-0.02	-0.09
	**Mg (mg Kg^{-1})**	-0.66	-0.59	0.43	-0.30	0.18	-0.11	-0.69	-0.36	0.31	0.24	-0.64	-0.20	-0.19	-0.60	-0.05	-0.68	0.30	-0.01	-0.61	-0.71	0.60	0.68	0.69	-0.19	1.00	0.29	0.05	-0.61	-0.64	-0.48
	**K (mg Kg^{-1})**	-0.07	-0.02	0.32	0.11	-0.41	0.34	-0.09	-0.30	-0.35	-0.18	0.06	-0.10	-0.27	-0.19	0.32	0.24	0.09	-0.38	0.03	-0.40	0.78	0.83	-0.08	-0.26	0.29	1.00	0.89	-0.72	-0.75	0.09
	**Na (mg Kg^{-1})**	-0.02	-0.03	0.19	-0.11	-0.53	0.16	0.04	-0.35	-0.21	-0.09	0.03	-0.25	-0.46	-0.14	0.40	0.38	-0.26	-0.19	0.06	-0.08	0.54	0.57	-0.28	-0.50	0.05	0.89	1.00	-0.47	-0.43	0.21
	**C:N ratio**	0.09	-0.02	-0.19	-0.22	0.18	-0.55	0.29	-0.07	0.01	0.45	0.08	0.20	0.10	0.21	-0.35	-0.11	-0.27	0.58	-0.10	0.56	-0.99	-0.87	-0.33	0.33	-0.61	-0.72	-0.47	1.00	0.75	-0.10
	**N:P ratio**	0.23	0.12	-0.61	-0.07	-0.02	-0.24	0.34	0.39	0.24	0.04	0.00	-0.14	-0.04	0.26	-0.05	0.14	-0.41	0.36	0.34	0.78	-0.77	-0.95	-0.19	-0.02	-0.64	-0.75	-0.43	0.75	1.00	0.32
	**MAP (mm)**	0.58	0.60	-0.58	0.38	-0.60	0.62	0.47	0.46	-0.38	-0.65	0.35	-0.18	-0.04	0.32	0.22	0.71	-0.18	-0.37	0.89	0.71	0.14	-0.21	-0.54	-0.09	-0.48	0.09	0.21	-0.10	0.32	1.00

Table S6. Correlation coefficients for the modelled variables (*indicates significant correlation).

	Quadratic diameter	Aboveground biomass	Stem density	Growth rate	Mortality rate	Aboveground wood productivity
**Quadratic diameter**	1	0.689*	-0.624*	-0.065	0.121	0.019
**Aboveground biomass**	0.689*	1	0.059	-0.392*	-0.197	-0.114
**Stem density**	-0.624*	0.059	1	-0.364*	-0.418*	-0.154
**Growth rate**	-0.065	-0.392*	0.364*	1	0.594*	0.749*
**Mortality rate**	0.121	-0.197	-0.418*	0.594*	1	0.428*
**Aboveground wood productivity**	0.019	-0.114	-0.154	0.749*	0.428*	1
